# Through Its Powerful Antioxidative Properties, L-Theanine Ameliorates Vincristine-Induced Neuropathy in Rats

**DOI:** 10.3390/antiox12040803

**Published:** 2023-03-25

**Authors:** Chih-Chuan Yang, Mao-Hsien Wang, Hung-Sheng Soung, Hsiang-Chien Tseng, Feng-Huei Lin, Kuo-Chi Chang, Cheng-Chia Tsai

**Affiliations:** 1 Department of Neurosurgery, Mackay Memorial Hospital, Taipei 10449, Taiwan; chetyang@gmail.com; 2 Department of Nursing, Mackay Junior College of Medicine, Nursing and Management, Taipei 11260, Taiwan; 3 Department of Anesthesia, En Chu Kon Hospital, Sanshia District, New Taipei City 23702, Taiwan; 4 Department of Psychiatry, Yuan-Shan Br. of Taipei Veteran General Hospital, Yilan County 26604, Taiwan; 5 Department of Biomedical Engineering, National Defense Medical Center, Taipei 11490, Taiwan; 6 Department of Anesthesiology, Shin Kong Wu Ho-Su Memorial Hospital, Taipei 11101, Taiwan; 7 School of Medicine, Fu Jen Catholic University, New Taipei City 24205, Taiwan; 8 Institute of Biomedical Engineering, National Taiwan University, Taipei 10051, Taiwan; 9 Institute of Biomedical Engineering and Nanomedicine, National Health Research, Zhunan Town, Miaoli County 35053, Taiwan; 10 Institute of Taiwan Instrument Research, National Applied Research Laboratories, Hsinchu 300092, Taiwan; 11 Department of Chemical Engineering and Biotechnology, National Taipei University of Technology, Taipei 10608, Taiwan; 12 Department of Medicine, Mackay Medical College, New Taipei City 252, Taiwan

**Keywords:** antioxidation, Vincristine, L-theanine, neuropathy

## Abstract

L-theanine (LT), which is a major amino acid found in green tea, was shown to alleviate Vincristine (VCR)-induced peripheral neuropathy and associated neuronal functional changes in rats. To induce peripheral neuropathy, rats were administered VCR at a dose of 100 mg/kg/day intraperitoneally on days 1–5 and 8–12, while control rats received LT at doses of 30, 100, and 300 mg/kg/day intraperitoneally for 21 days or saline solution. Electrophysiological measurements were taken to evaluate the nerve functional loss and recovery through motor and sensory nerve conduction velocities. The sciatic nerve was examined for several biomarkers, including nitric oxide (NO), malondialdehyde (MDA), glutathione (GSH), superoxide dismutase (SOD), catalase (CAT), total calcium, IL-6, IL-10, MPO, and caspase-3. The results showed that VCR caused significant hyperalgesia and allodynia in rats; decreased nerve conduction velocity; increased NO and MDA levels; and decreased GSH, SOD, CAT, and IL-10 levels. LT was found to significantly reduce VCR-induced nociceptive pain thresholds, decrease oxidative stress levels (NO, MDA), increase antioxidative strength (GSH, SOD, CAT), and reduce neuroinflammatory activity and apoptosis markers (caspase-3). LT’s antioxidant, calcium homeostasis, anti-inflammatory, anti-apoptotic, and neuroprotective properties make it a potential adjuvant to conventional treatment in VCR-induced neuropathy in rats.

## 1. Introduction

Chemotherapy-induced neuropathic pain is a prevalent adverse effect of cancer treatment caused by various chemotherapeutic medications, such as vinca alkaloids, platinum medicines, and taxanes [1]. This condition affects approximately 30–40% of patients worldwide and persists even after treatment cessation, resulting in the reduced efficacy of anti-cancer treatments and a decline in the quality of life [2]. Among the vinca alkaloids, Vincristine (VCR), which is derived from the Madagascar Catharanthus roseus plant, is a primary antineoplastic agent used to treat lymphomas, leukemias, and sarcomas. VCR works by inhibiting tubulin polymerization and microtubule incorporation, ultimately preventing mitotic spindle assembly and causing mitosis [3]. However, VCR administration leads to various nociceptive pain behaviors, such as hyperalgesia, dysesthesia, and allodynia, which are severe dose-limiting side effects that require treatment discontinuation, thereby putting cancer patients’ lives at risk and incurring significant healthcare costs [3,4]. Despite the high prevalence of VCR-induced neuropathic pain, there is a lack of appropriate preventive and therapeutic strategies due to the variable symptoms and poor understanding of the etiology and pathogenesis of this condition [3,4].

Conventional analgesics and antineuralgic drugs provide limited relief and are restricted due to side effects [2,3,4]. Therefore, there is a need to explore novel therapeutic techniques for neuropathy in medical research. The rodent model of VCR-induced neuropathy is commonly used to study neuropathic pain syndrome, as it closely resembles clinical findings in human patients with cancer-chemotherapy-induced neuropathic pain syndromes [4,5,6,7,8]. Although the specific mechanism of VCR-induced neuropathy synthesis is unknown, recent studies highlighted various mechanisms, including nitrosative and oxidative stress and neuroinflammation. Pharmacological studies using this model suggested that well-known antioxidant or anti-inflammatory drugs effectively treat VCR-induced neuropathy in animal models [3,4,5,6,7,8]. Therefore, these drugs may be potential candidates for neuropathy therapy by decreasing nitrosative and oxidative stress and inflammation.

L-theanine (LT) is a molecule with a similar structure to glutamate and glutamine, accounting for 0.5–2% of the dry weight of tea leaves. LT consists of ethylamine and glutamate and is rapidly absorbed into the circulation of the gastrointestinal system and subsequently distributed to various organs, including the brain, via cotransport with Na^+^ [9]. LT was reported to have taste-enhancing properties and health benefits and has been widely utilized in the food and pharmaceutical industries, as it does not produce any toxic effects in animals or humans according to toxicological and technical reports [9,10]. Additionally, LT was shown to possess sedative, hypotensive, anti-obesogenic, and anti-inflammatory properties [9,10,11], as well as the ability to scavenge reactive radicals and mitigate peroxidative conditions. LT was proposed to have neuroprotective properties against behavioral deficits resulting from toxins, stress, and spinal cord nerve injury [12,13,14,15]. However, the effects of LT on peripheral neuropathy caused by VCR and its underlying mechanisms remain largely unknown. Therefore, this study aimed to investigate whether LT can alleviate VCR-induced neuropathy in a rat model that mimics the signs and symptoms of human neuropathic pain and whether it can mitigate nitrosative/oxidative stress and neuroinflammation.

In this study, we aimed to investigate the potential of LT in alleviating VCR-induced neuropathy in rats using a well-established model. Various assessments were carried out, including the Von Frey hair test, acetone drop test, and hot plate test to evaluate the degree of neuropathy. Additionally, electrophysiological investigations were conducted to examine sensory and motor nerve conduction velocities (SNCV and MNCV). The levels of nitrite (NO) and malondialdehyde (MDA) were measured to determine the nitrosative and oxidative statuses, while glutathione (GSH), superoxide dismutase (SOD), and catalase (CAT) enzyme activities were analyzed to evaluate the antioxidant power. Total calcium, tumor necrosis factor-α (TNF-α), interleukin-6 (IL-6), interleukin-10 (IL-10), myeloperoxidase, and severe neurodegeneration were also examined, as they were strongly associated with VCR-induced neuropathy in animal models [3,4,5,6,7,8].

## 2. Materials and Methods

### 2.1. Animals

Male Wistar rats weighing between 220 and 250 g (obtained from BioLASCO Taiwan Co., Ltd., Taipei, Taiwan) were utilized in this study. The animals were housed in Plexiglas cages in groups of three with ad libitum access to food and water under controlled temperature conditions (22 ± 3 °C) and a 12 h light/dark cycle with the lights turned on at 7:00 a.m. All study procedures were approved by the Institutional Animal Care and Use Committee (IACUC) of the National Taiwan University College of Medicine and College of Public Health, following the guidelines published by the National Institutes of Health of the United States (IACUC approval no. 20180183). The study tests were conducted during the light phase of the cycle, and the animals were euthanized using a CO_2_ overdose at the end of the study.

### 2.2. Drugs

Physiological saline and distilled water were used to prepare Vincristine sulfate salt (VCR; V8879-5MG; Sigma, St. Louis, MO, USA) and N-ethyl-L-glutamine (LT ≥ 98%; SMB00395-500MG; Sigma, USA) for the study. The doses used in the study were based on previous research [14]. LT was administered intraperitoneally, with a pretest starting at a low dose of 1 mg/kg, gradually increasing to a maximum of 1000 mg/kg to observe statistically significant effects. Therefore, doses of 30/100/300 mg/kg were used in the present study. Vincristine sulfate (100 μg/kg/day) was administered intraperitoneally for two 5-day cycles with a 2-day pause (days 1–5 and 8–12) to induce peripheral neuropathy [8].

### 2.3. Experimental Protocol

**Figure 1:**
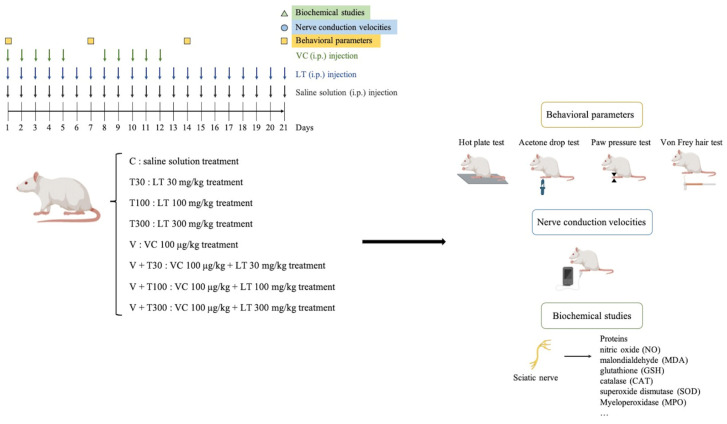
Summary of the animal experimental design. In this experiment, rats were randomly assigned to the following eight groups: C, T30, T100, T300, V, V + T30, V + T100, and V + T300.

For this study, male Wistar rats were randomly divided into eight groups with eight rats in each group: control (C), LT 30 mg/kg treatment (T30), LT 100 mg/kg treatment (T100), LT 300 mg/kg treatment (T300), VCR treatment (V), VCR + LT 30 mg/kg treatment (V + T30), VCR + LT 100 mg/kg treatment (V + T100), and VCR + LT 300 mg/kg treatment (V + T300). The control group received an intraperitoneal (i.p.) injection of normal saline solution. The T30, T100, and T300 groups received i.p. injections of LT at doses of 30, 100, or 300 mg/kg, respectively, for 21 days. The V group received i.p. injections of 100 μg/kg VCR for two 5-day cycles with a 2-day pause (days 1–5 and 8–12) and normal saline i.p. on days 1–21. The V + T30, V + T100, and V + T300 groups received i.p. injections of 100 μg/kg VCR for two 5-day cycles with a 2-day pause (days 1–5 and 8–12) and LT i.p. at doses of 30, 100, or 300 mg/kg, respectively, on days 1–21. VCR was administered 60 min before the LT or normal saline injection on days 1–5 and 8–12. Behavioral parameters were measured before the VCR administration on day 1 and days 7, 14, and 21 after each LT or normal saline injection. Nerve conduction velocities were measured after the behavioral examinations on day 21 for electrophysiological studies. All biochemical studies were conducted after the electrophysiological experiments were completed (see Figure 1). During the study, eight rats died naturally for unknown reasons.

### 2.4. Behavioral Assessment

#### 2.4.1. Hot Plate Test

We evaluated the pain sensitivity of the hind paws of rats for thermal hyperalgesia using Eddy’s hot plate (Sorel Hot Plate model DS37, Ugo Basile, Gemonio, Italy), as per the method described by Eddy et al. [16], with certain modifications for measuring the intensity of noxious thermal sensation. The rats were placed on a hot plate surface that was preheated to 52.5 ± 0.5 degrees Celsius and allowed to withdraw their left hind paw to the extent of their nociceptive threshold. A 20 s cut-off time was strictly observed.

#### 2.4.2. Acetone Drop Test

To evaluate the sensitivity of rat hind paws to chemical allodynic pain, we used an acetone drop application following the protocol of Yoon et al. [17], with a slight adaptation to assess the reactivity to non-noxious cold chemical stimuli. Rats were placed on a wire mesh grid, allowing access to their hind paws. We sprayed 100 mL of acetone (W332615-100G; Sigma, USA) onto the left hind paw of each rat. We recorded paw licking, shaking, or rubbing as a paw withdrawal threshold in response to cold chemical exposure, with a cut-off time of 20 s.

#### 2.4.3. Paw Pressure Test

By increasing the pressure on the left hind paw, we measured the mechanical nociceptive threshold in grams (Coulbourn Instruments LLC, Holliston, MA, USA). The mechanical nociceptive threshold was determined by withdrawing the left hind paw. The cutoff pressure was kept at 450 g [18].

#### 2.4.4. Von Frey Hair Test

The mid-plantar surface of the left hind paw was tested for mechanical allodynia using calibrated nylon filaments (Linton Instrumentation, Norfolk, UK) of varying stiffnesses. Each filament was applied to the paw 10 times, beginning with the softest and increasing in stiffness. A positive response was recorded if the left hind limb was quickly withdrawn. To determine the threshold value in grams, the filament was applied five times out of ten trials (50% response). A cut-off pressure of 30 g was used [19].

### 2.5. Electrophysiological Studies

After assessing various behavioral parameters on day 21, the animals were anesthetized for electrophysiological studies of motor and sensory nerve conduction velocities.

#### 2.5.1. Motor Nerve Conduction Velocity Assessment

The student physiograph (INCO Pvt. Ltd., Ambala, India) and biopotential coupler and stimulator device were used to evaluate the motor nerve conduction velocity (MNCV) as per Thomsen et al. [20] and Saini et al. [21] with minor modifications. On day 21, anesthesia was induced via intravenously injecting 300 mg/kg of chloral hydrate. Bipolar needle electrodes (26 1/2-gauge) were used to apply a supramaximal stimulus (3 V single stimulus and squarewave pulses for a 0.2 ms duration) to stimulate the sciatic (proximally to the sciatic notch) and tibial nerves (distally to the ankle). Paired metal needle electrodes were inserted percutaneously across the stimulation or recording electrode and the reference electrode. The electrodes were placed over the small muscles of the hind paw dorsum, and an alligator clip was used to attach a ground electrode to the calf muscle. Electrical stimulation was applied to the knee and sciatic notch to detect the motor response. Rats were maintained at a body temperature of 37 °C during the study. To record the motor fiber stimulation, a student physiograph was used. The latency (from the point of stimulation to the onset of response) was measured in milliseconds, and the distance between the two electrodes was measured in millimeters. The motor nerve conduction velocity was calculated using the formula: motor nerve conduction velocity = (distance between the nerve stimulation point)/(latency period difference).

#### 2.5.2. Sensory Nerve Conduction Velocity Determination

A modified technique, as described by Kurokawa et al. [22], was utilized to record the sensory nerve conduction velocity (SNCV) using a student physiograph and biopotential coupler. An active electrode was positioned 1 cm proximal to a reference electrode, which was situated behind the medial malleolus at the ankle. A square-wave pulse with a duration of 0.05 ms was administered at a rate of 1 H_Z,_ and 10 pulses per train of intensity current were applied to the middle (3rd) digital nerve of the hind leg, resulting in a maximum amplitude response. The peak-to-peak amplitude was subsequently measured. The recording needles were maintained at a distance of 10 mm apart, and there was an approximate 25 mm separation between the recording and stimulation electrodes. The body temperature of the rats remained at a constant 37 °C throughout the study. Latencies were measured during the stimulus–response interval, and the maximal sensory nerve action potential was calculated using the following formula: the sensory nerve conduction velocity, also known as the H-reflex, was determined by dividing the onset of latency by the peak of the initial negative deflection (distance between stimulating and recording electrodes).

### 2.6. Biochemical Estimations

On day 21, following the completion of electrophysiological studies, a CO_2_ chamber was utilized to minimize stress. Both legs’ sciatic nerves were immediately isolated, and the nerve was excised distal and proximal to the point of transection. Exactly beneath the point of transection of the sciatic nerve, a 1 cm diameter piece of tissue was removed. The samples were frozen and subsequently analyzed together after freezing. A sciatic nerve homogenate (10%, *w*/*v*) was prepared using 0.1 M tris HCl buffers (pH 7.4). The homogenates were kept in ice water for 30 min, followed by centrifugation at 2000× *g*. Proteins, nitric oxide (NO), malondialdehyde (MDA), glutathione (GSH), catalase (CAT), and superoxide dismutase (SOD) were measured from the clear supernatant. The tissue beneath the sciatic nerve was homogenized and centrifuged at 5000× *g* for 10 min. The myeloperoxidase (MPO) activity was subsequently measured in pellets following centrifugation.

#### 2.6.1. Total Protein Content

The protein concentration was estimated using the method developed by Lowry et al. [23], with bovine serum albumin (obtained from Sigma, USA) as the standard. The absorbance was measured at 750 nm using spectrophotometry.

#### 2.6.2. Measurement of Oxidative Stress

##### Nitrite Concentration

To determine the oxidative status of nitrites in the sciatic nerve samples, 150 μL of the supernatant was dissolved in 0.3 M sodium hydroxide. After 5 min, the precipitated protein was removed by adding 75 μL of zinc sulfate (5%). The mixture was subsequently centrifuged at 15,000× *g* for 20 min at 4 °C to separate the supernatant. Next, 300 μL of vanadium (III) chloride (80 mg VCl_3_ in 10 mL HCl, 1 M; Sigma, USA) was added to 200 μL of the supernatant. The mixture was incubated at 37 °C for 45 min. The absorbance was measured at a wavelength of 540 nm. A standard curve was generated using sodium nitrite [24]. The results were expressed as nmol/mg of protein.

##### Malondialdehyde

To assess the tissue lipid peroxidation levels, the concentration of MDA was determined by reacting it with TBA, following the procedure outlined in [25]. In short, 150 μL of supernatant was combined with 300 μL of 20% trichloroacetic acid (obtained from Sigma, USA) and 0.67% TBA (obtained from Sigma, USA). The mixture was heated in boiling water for 60 min and then allowed to cool to room temperature. Next, the samples were centrifuged at 3500× *g* for 10 min. The resulting supernatant was then measured for its absorbance at 532 nm, and standard curves were created using Tetramethoxypropane (obtained from Sigma, USA). The concentration of MDA was expressed as nmol/mg protein.

##### Glutathione Concentration

To measure the reduced glutathione concentration, equal amounts of sciatic nerve homogenate were mixed with 10% trichloroacetic acid (Sigma, USA) and centrifuged to separate the proteins. Then, 2 milliliters of phosphate buffer (pH 8.4), 500 milliliters of 2,5-dinitrobenzoic acid, and 400 milliliters of double-distilled water were added to 10 microliters of the resulting supernatant. The mixture was vortexed and the absorbance was measured at 412 nm within 15 min. The concentration of reduced glutathione was reported as μg/mg protein, according to the method described in [26].

##### Superoxide Dismutase Activity

To measure the superoxide dismutase (SOD) activity, xanthine-xanthine oxidase (Sigma, USA) was used as a superoxide generator to inhibit the reduction of nitro blue tetrazolium. A 5/3 *v*/*v* mixture of ethanol and chloroform was added to the same volume of sample and centrifuged, and the activity was measured in the ethanol phase of the supernatant. One unit of SOD was defined as the amount required to inhibit 50% of the reduction of nitro blue tetrazolium. The SOD activity was expressed as U/mg protein, according to the method described in [27].

##### Catalase Activity

The catalase activity was determined by measuring the absorbance of hydrogen peroxide (H_2_O_2_; Sigma, USA) at 240 nm, according to the method of Aebi [28]. The catalase activity was evaluated via the decomposition of 1 μmol of hydrogen peroxide per minute (μM (H_2_O_2_)/min) at 25 °C, which produced lysate by catalysis. Bovine serum albumin was used as the standard to determine the total protein concentration in the cell lysates [29]. The results were expressed as U/mg protein.

#### 2.6.3. Total Calcium

The total calcium was estimated by mixing sciatic nerve tissue homogenate with 1 mL of 4% trichloroacetic acid (Sigma, USA) in ice-cold conditions, followed by centrifugation at 2000× *g* for 10 min. The clear supernatant was analyzed for total calcium ions using atomic emission spectroscopy at 556 nm. The results were expressed as ppm/mg of protein, according to the method described in [30].

#### 2.6.4. Measurement of Neuroinflammatory Markers

##### Interleukin-6, Interleukin-10, and Tumor Necrosis Factor-α

To estimate the levels of interleukin-6 (IL-6), interleukin-10 (IL-10), and tumor necrosis factor-α (TNF-α) in the nerve samples, the samples were first homogenized in 0.40 mL of ice-cold phosphate buffer saline and centrifuged at 10,000× *g* for 15 min, following the procedure outlined in [31]. The resulting supernatant was then used to measure IL-6, IL-10, and TNF-α through an enzyme-linked immunosorbent assay (ELISA) using ultra-sensitive ELISA kits (obtained from Ray Biotech Inc., Peachtree Corners, GA, USA) and following the manufacturer’s instructions. The concentrations of cytokines were determined via interpolation using standard curves assayed on individual plates, and the results were expressed as pg/mL protein.

##### Myeloperoxidase

The activity of myeloperoxidase (MPO) was determined by reading the standards at 460 nm on a spectrophotometer. One unit of MPO activity was defined as the change in absorbance of 1.0 unit/min at pH 7.0 and a temperature of 25 °C and was calculated from the initial reaction rate with peroxide (1 μM) as the substrate. The results were expressed as U/mg of protein [32].

#### 2.6.5. Measurement of the Apoptosis Marker

##### Caspase-3 Activity

The levels of caspase-3, which is an executioner molecule in the apoptotic cascade, were determined by cleaving the chromogenic caspase substrate Ac-DEVD-pNA, which is a protease that is rapidly activated when cells are exposed to apoptotic conditions and cleaves poly(ADP-ribose) polymerase. The amount of caspase-3 was measured at 405 nm using a spectrophotometer following the manufacturer’s instructions (R&D Systems, BiosPacific, Emeryville, CA, USA). The results were expressed as ng/mg protein.

### 2.7. Statistical Analysis

The mean ± standard error of the mean was used to represent all the results. A repeated-measure three-way analysis of variance (ANOVA) was used to analyze the behavioral assessment data. A two-way ANOVA was used to analyze the electrophysiological data and biochemical estimations separately. Tukey’s test was used to compare groups post hoc. Statistical significance was set at *p* < 0.05.

## 3. Results

The values of the tested parameters were very similar before the VCR administration. The post hoc analysis of the tested parameters showed no significant differences between the LT groups (30, 100, or 300 mg/kg; T30, T100, or T300) and the C groups (T30 vs. C, *p* > 0.05; T100 vs. C, *p* > 0.05; T300 vs. C, *p* > 0.05).

### 3.1. Effect of LT on the Development of Thermal Hyperalgesia and Allodynia Induced by VCR

**Figure 2:**
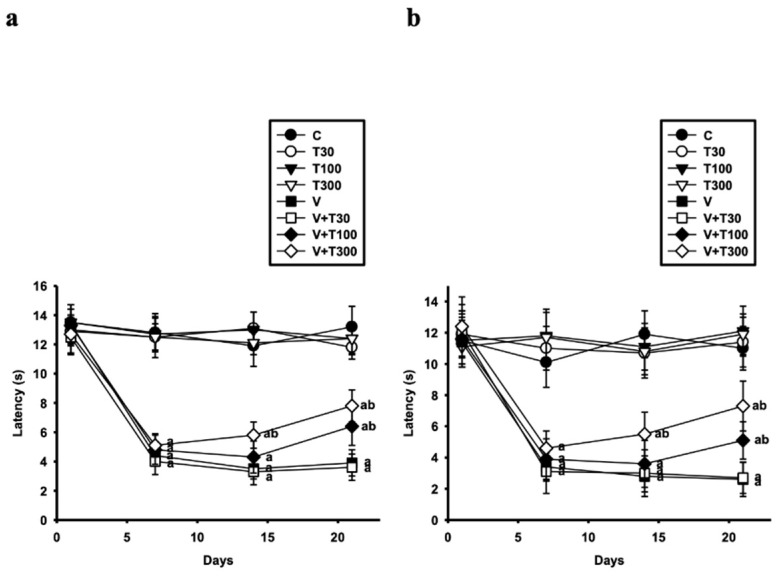
The effects of LT on VCR-induced neuropathy behavioral characteristics as shown in the hot plate test (**a**) and acetone drop test (**b**). Data were analyzed using repeated and three-way ANOVA with Tukey’s pairwise tests and expressed as the mean ± SD, *n* = 8 per group. ^a^ *p* < 0.001 compared with C; ^b^ *p* < 0.001 compared with V.

VCR-induced neuropathy; caused significant development in thermal/mechanical hyperalgesia and allodynia. To investigate the ameliorative effects of LT on VCR-induced neuropathy in rats, LT was administered. VCR-induced neuropathy led to the production of persistent thermal hyperalgesia and allodynia with a reduction in response latency at approximately day 7, with this effect peaking on day 14 and lasting through to the end of the experiment for the hot plate test (Figure 2a) (7 days: –66%; 12.8 ± 1.3–4.4 ± 0.5 s) (*p* < 0.001) (14 days: −71%; 11.9 ± 1.4–3.5 ± 0.7 s) (*p* < 0.001) (21 days: −70%: 13.2 ± 1.4–3.9 ± 0.9 s) (*p* < 0.001) and acetone drop test (Figure 2b) (7 days: –66%; 10.1 ± 1.6–3.4 ± 0.9 s) (*p* < 0.001) (14 days: −76%; 11.9 ± 1.5–2.8 ± 1 s) (*p* < 0.001) (21 days: −76%: 11 ± 1.4–2.6 ± 1.1 s) (*p* < 0.001) compared with the C group.

No significant changes were found in the response latency in the V + T30 group (7 days, *p* > 0.05; 14 days, *p* > 0.05; 21 days, *p* > 0.05) compared with the V group in the hot plate and acetone drop tests. However, LT (100 mg/kg) significantly increased the response latency, but only on day 21 of treatment compared with the V group in the hot plate (3.9 ± 0.9–6.4 ± 1.3 s) (*p* < 0.001) and acetone drop tests (2.6 ± 1.1–5.1 ± 1.2 s) (*p* < 0.001). In addition, LT (300 mg/kg) increased the response latency significantly in rats compared with the V group on day 14 of treatment in the hot plate and acetone drop tests (hot plate test: 3.5 ± 0.7–5.8 ± 0.9 s) (*p* < 0.001) (acetone drop test: 2.8 ± 1–5.5 ± 1.4 s) (*p* < 0.001) and on day 21 (hot plate test: 3.9 ± 0.9–7.8 ± 1.1 s) (*p* < 0.001) (acetone drop test: 2.6 ± 1.1–7.3 ± 1.6 s) (*p* < 0.001). These results indicate that the LT treatment prevented the VCR-induced development of thermal hyperalgesia and allodynia.

### 3.2. Effect of LT on the Development of Mechanical Hyperalgesia and Allodynia Induced by VCR

**Figure 3:**
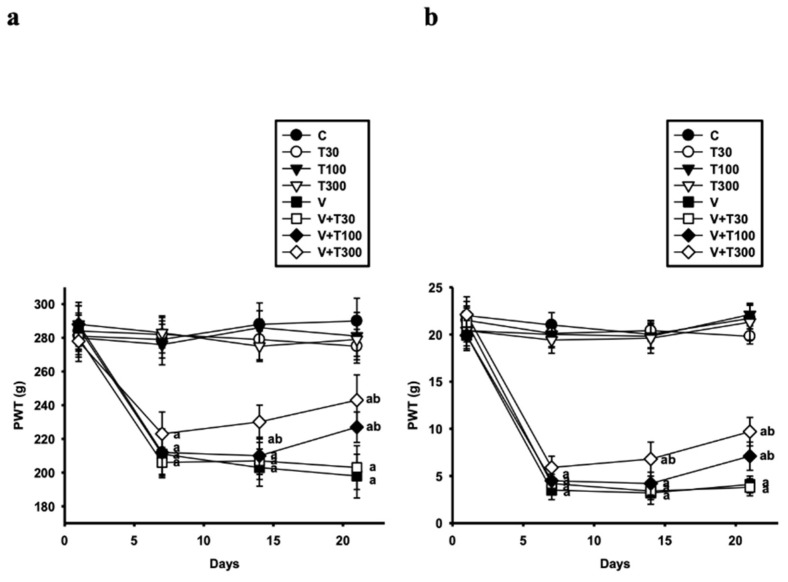
The effects of LT on the VCR-induced neuropathy behavioral characteristics in the paw pressure test (**a**) and Von Frey hair test (**b**). Data were analyzed using repeated and three-way ANOVA with Tukey’s pairwise tests and expressed as the mean ± SD, *n* = 8 per group. ^a^ *p* < 0.001 compared with C; ^b^ *p* < 0.001 compared with V.

On day 7, the VCR administration also caused significant development of mechanical hyperalgesia and allodynia as shown by a decrease in the hind paw withdrawal threshold (PWT) in the paw pressure test (Figure 3a) (7 days: −24%; 279 ± 11–211 ± 12 g) (*p* < 0.001) and von Frey hair test (Figure 3b) (7 days: −83%; 21 ± 1.3–3.5 ± 1 g) (*p* < 0.001) in the V group. The low PWT of the V group was maintained throughout the study in the paw pressure test (14 days: −30%; 288 ± 12.7–203 ± 11 g) (*p* < 0.001) (21 days: −32%; 290 ± 13.5–198 ± 13 g) (*p* < 0.001) and von Frey hair test (14 days: −84%; 20 ± 1.4–3.2 ± 1.2 g) (*p* < 0.001) (21 days: −81%; 21.7 ± 1.4–4.1 ± 0.9 g) (*p* < 0.001).

There were no significant differences in the PWT (7 days, *p* > 0.05; 14 days, *p* > 0.05; 21 days, *p* > 0.05) between the V + T30 and V groups. However, the administration of LT (100 and 300 mg/kg) attenuated the VCR-induced decrease in the PWT for mechanical hyperalgesia (14 days—LT 300 mg/kg: 203 ± 11–230 ± 10 g) (V vs. V + T300, *p* < 0.001) (21 days—LT 100 mg/kg: 198 ± 13–227 ± 9 g and LT 300 mg/kg: 198 ± 13–243 ± 15 g) (V vs. V + T100, *p* < 0.001 and V vs. V + T300, *p* < 0.001) and allodynia (14 days—LT 300 mg/kg: 3.2 ± 1.2–6.8 ± 1.8 g) (V vs. V + T300, *p* < 0.001) (21 days—LT 100 mg/kg: 4.1± 0.9–7.1 ± 1.5 g and LT 300 mg/kg: 4.1 ± 0.9–7.5 ± 1.5 g) (V vs. V + T100, *p* < 0.001 and V vs. V + T300, *p* < 0.001) compared with the V group in a dose-dependent manner from day 14 to 21 after the treatment. These results indicate that the LT treatment prevented the VCR-induced development of mechanical hyperalgesia and allodynia. Based on the above results, our observed NP was verified since the LT treatment ameliorated VCR-induced neuropathy.

### 3.3. Effect of LT on the Loss of Neuronal Electrical Function Induced by VCR

**Figure 4:**
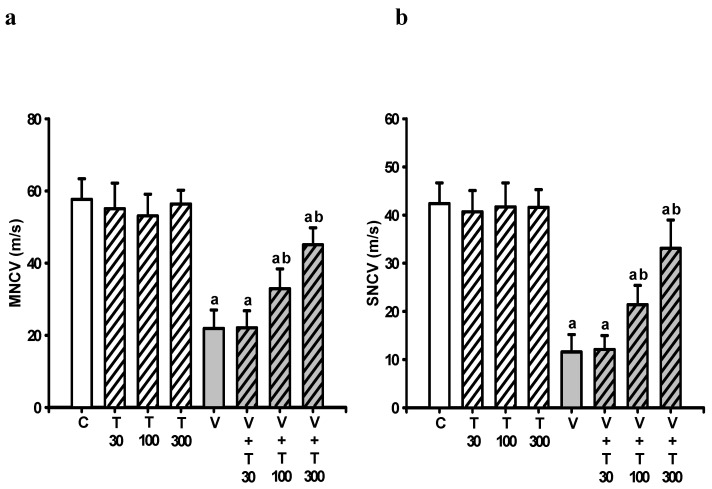
The effects of LT on VCR-induced neuropathy electrophysiological characteristics in terms of the motor nerve conduction velocity (MNCV) (**a**) and sensory nerve conduction velocity (SNCV) (**b**). Data were analyzed using repeated and three-way ANOVA with Tukey’s pairwise tests and expressed as the mean ± SD, *n* = 8 per group. ^a^ *p* < 0.001 compared with C; ^b^ *p* < 0.001 compared with V.

To investigate the effects of VCR and the ameliorative role of LT on the neuronal electrical function in rats, the MNCV and SNCV in the rat sciatic nerve were examined. The VCR administration resulted in a loss of neuronal electrical function, as indicated by the decrease in the MNCV (57.7 ± 5.7–21.9 ± 5.1 m/s; −62%) (C vs. V, *p* < 0.001) (Figure 4a) and SNCV (42.4 ± 4.3–11.6 ± 3.6 m/s; −73%) (C vs. V, *p* < 0.001) (Figure 4b) on day 21, suggesting that their myelinated nerve fibers were damaged.

The LT administration (100 and 300 mg/kg) attenuated the VCR-induced decreases in MNCV (LT 100 mg/kg: 21.9 ± 5.1–32.9 ± 5.4 m/s (31%); LT 300 mg/kg: 21.9 ± 5.1–45.1 ± 4.7 m/s (65%)) (V vs. V + T100, *p* < 0.001; V vs. V + T300, *p* < 0.001) and SNCV (LT 100 mg/kg: 11.6 ± 3.6–21.4 ± 3.9 m/s (32%); LT 300 mg/kg: 11.6 ± 3.6–33.1 ± 5.9 m/s (70%)) (V vs. V + T100, *p* < 0.001; V vs. V + T300, *p* < 0.001) in a dose-dependent manner. These results indicate that the LT treatment prevented a VCR-induced loss of neuronal electrical function.

### 3.4. Effect of LT on the Increases in Sciatic Nitric Oxide and Lipid Peroxide Production Induced by VCR

**Figure 5:**
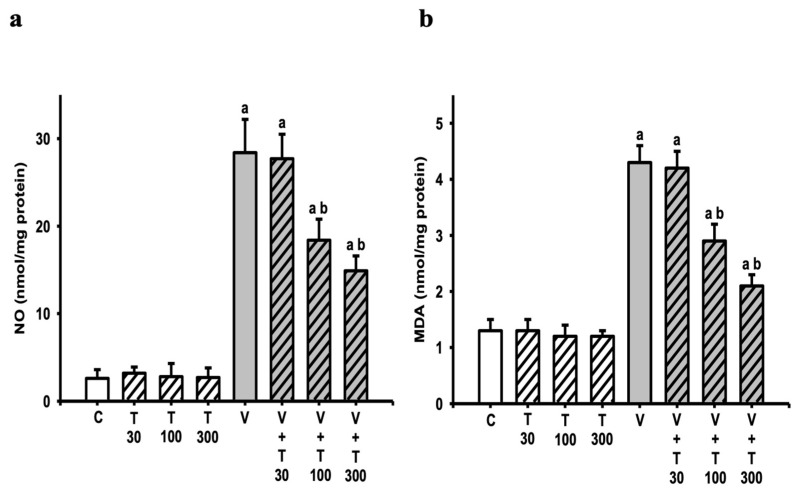
The effects of LT on VCR-induced dysfunctions in terms of the levels of nitrate (**a**) and MDA (**b**) in rat sciatic nerves. Data were analyzed using repeated and three-way ANOVA with Tukey’s pairwise tests and expressed as the mean ± SD, *n* = 8 per group. ^a^ *p* < 0.001 compared with C; ^b^ *p* < 0.001 compared with V.

In this study, we examined the levels of nitrite (NO), malondialdehyde (MDA), glutathione (GSH), superoxide dismutase (SOD), and catalase (CAT) enzyme activities in the sciatic nerve. On day 21, compared with the control (C) group, both the nitrite (2.6 ± 1–28.4 ± 3.8 nmol/mg protein (992%)) (*p* < 0.001) (Figure 5a) and MDA (1.3 ± 0.2–4.3 ± 0.3 nmol/mg protein (231%)) (*p* < 0.001) (Figure 5b) levels were significantly increased in the rat sciatic nerves of the V group.

However, the treatment with LT at doses of 100 mg/kg and 300 mg/kg significantly inhibited the elevated nitrite and MDA levels in the V groups (nitrite: 28.4 ± 3.8–18.4 ± 2.4 nmol/mg protein (−39%) and MDA: 4.3 ± 0.3–2.9 ± 0.3 nmol/mg protein (−47%)) (V vs. V + T100, nitrite: *p* < 0.001 and MDA: *p* < 0.001) (nitrite: 28.4 ± 3.8–14.9 ± 1.7 nmol/mg protein (−52%) and MDA: 4.3 ± 0.3–2.1 ± 0.2 nmol/mg protein (−73%)) (V vs. V + T300, nitrite: *p* < 0.001 and MDA: *p* < 0.001). These results suggest that the LT treatment could effectively inhibit VCR-induced increases in sciatic nitric oxide and lipid peroxide production.

### 3.5. Effect of LT on the Decreases in Sciatic Antioxidation Power Induced by VCR

**Figure 6:**
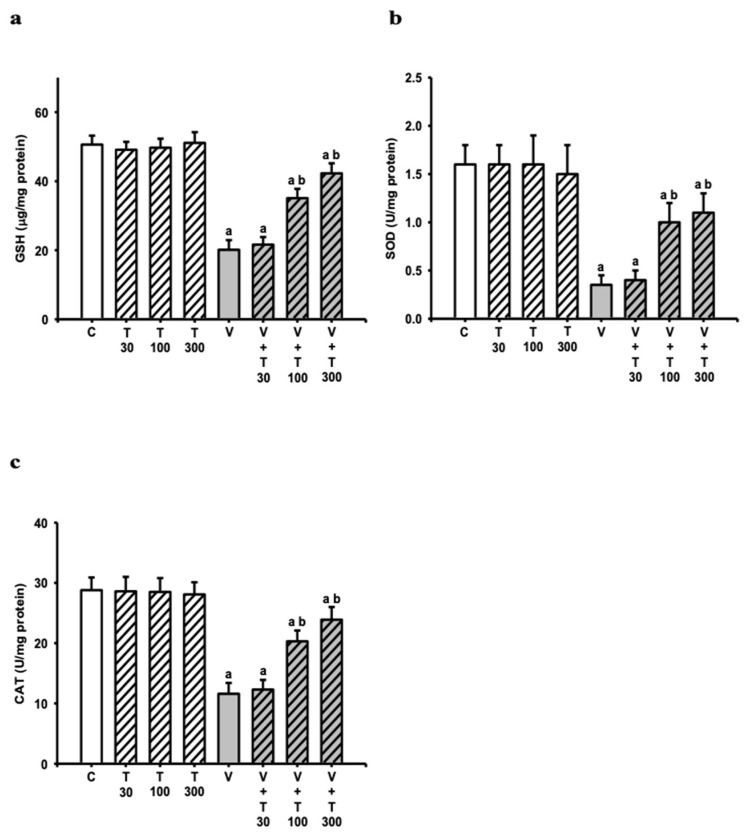
The effects of LT on VCR-induced dysfunctions in terms of the levels of GSH (**a**), SOD (**b**), and CAT(**c**) in rat sciatic nerves. Data were analyzed using repeated and three-way ANOVA with Tukey’s pairwise tests and expressed as the mean ± SD, *n* = 8 per group. ^a^ *p* < 0.001 compared with C; ^b^ *p* < 0.001 compared with V.

The levels of antioxidation power in the rat sciatic nerves were significantly decreased on day 21 compared with the C group, as indicated by the following changes: GSH (50.6 ± 2.6–20.1 ± 2.8 μg/mg protein (−60%)) (*p* < 0.001) (Figure 6a), SOD (1.6 ± 0.2–0.4 ± 0.1 U/mg protein (−75%)) (*p* < 0.001) (Figure 6b), and CAT (28.8 ± 2.1–11.6 ± 1.8 U/mg protein (−60%)) (*p* < 0.001) (Figure 6c).

However, the decreased levels of GSH, SOD, and CAT in the V groups were significantly restored after the LT 100 mg/kg treatment (GSH: 20.1 ± 2.8–35.1 ± 2.7 μg/mg protein (49%); SOD: 0.4 ± 0.1–1 ± 0.2 U/mg protein (50%); CAT: 11.6 ± 1.8–20.3 ± 1.8 U/mg protein (51%)) (V vs. V + T100, GSH: *p* < 0.001; SOD: *p* < 0.001; CAT: *p* < 0.001) and 300 mg/kg treatment (GSH: 20.1 ± 2–42.3 ± 2.9 μg/mg protein (73%); SOD: 0.4 ± 0.1–1.1 ± 0.2 U/mg protein (58%); CAT: 11.6 ± 1.8–23.9 ± 2.1 U/mg protein (72%)) (V vs. V + T300, GSH: *p* < 0.001; SOD: *p* < 0.001; CAT: *p* < 0.001). Therefore, the results suggest that the LT treatment prevented VCR-induced decreases in sciatic antioxidation power.

### 3.6. Effect of LT on the Increases in Sciatic Total Calcium Induced by VCR

**Figure 7:**
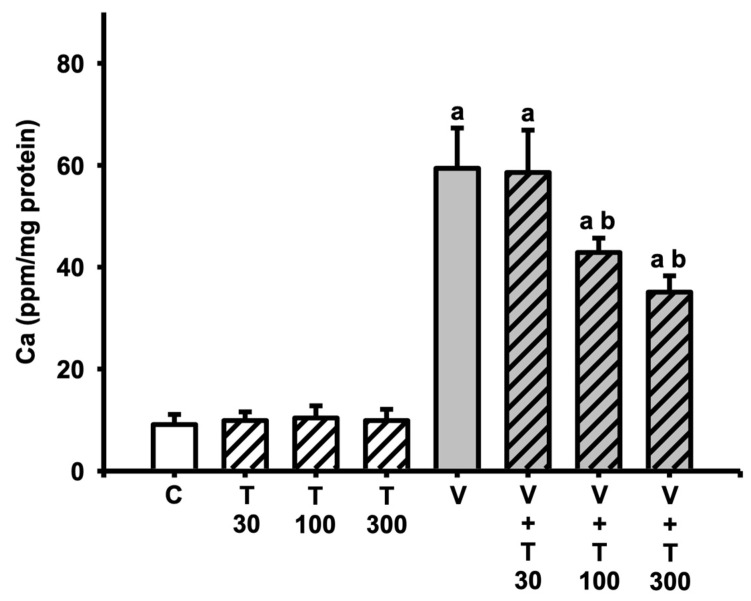
The effects of LT on VCR-induced dysfunctions in terms of the level of calcium in rat sciatic nerves. Data were analyzed using repeated and three-way ANOVA with Tukey’s pairwise tests and expressed as the mean ± SD, *n* = 8 per group. ^a^ *p* < 0.001 compared with C; ^b^ *p* < 0.001 compared with V.

In addition, the level of total calcium was also examined because the altered sciatic calcium level was correlated with VCR-induced neuropathy. The level of total calcium was also examined in the rat sciatic tissues. The total calcium level (9.1 ± 2–59.4 ± 7.9 ppm/mg of protein (553%)) (*p* < 0.001) (Figure 7) in the rat sciatic nerves were increased on day 21 compared with the C group.

The increased total calcium levels in the V groups were inhibited by the LT 100 mg/kg treatment (59.4 ± 7.9–42.9 ± 2.8 ppm/mg of protein (−33%)) (V vs. V + T100, total calcium: *p* < 0.001) and 300 mg/kg treatment (59.4 ± 7.9–35.1 ± 3.2 ppm/mg protein (−48%)) (V vs. V + T300, total calcium: *p* < 0.001). These results indicate that the LT treatment inhibited VCR-induced increases in sciatic total calcium.

### 3.7. Effect of LT on the Increases in Sciatic Neuroinflammatory and Apoptotic Markers Induced by VCR

**Figure 8:**
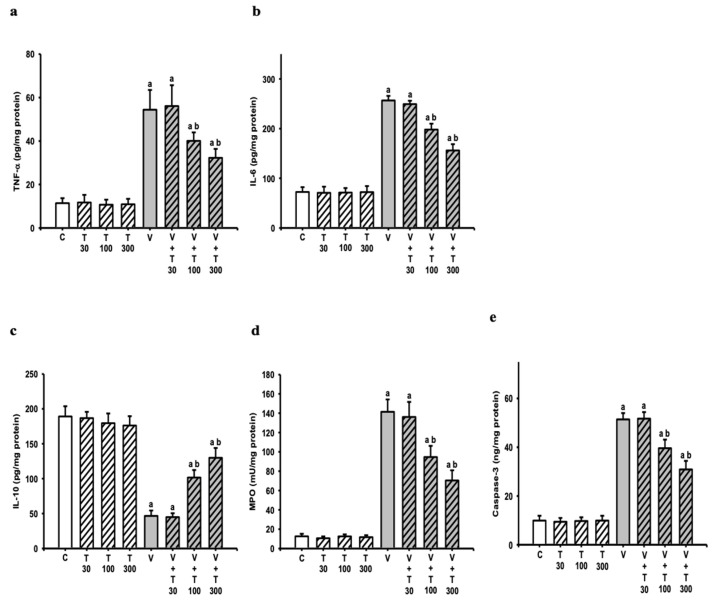
The effects of LT on VCR-induced dysfunctions in terms of the levels of TNF-α (**a**), IL-6 (**b**), IL-10 (**c**), MPO (**d**), and caspase-3 (**e**) in rat sciatic nerves. Data were analyzed using repeated and three-way ANOVA with Tukey’s pairwise tests and expressed as the mean ± SD, *n* = 8 per group. ^a^ *p* < 0.001 compared with C; ^b^ *p* < 0.001 compared with V.

Further, sciatic neuroinflammation and severe neurodegeneration were closely correlated with VCR-induced neuropathy. The levels of TNF-α, IL-6, IL-10, MPO, and caspase-3 were examined. Compared with the C groups, the sciatic levels of TNF-α (11.4 ± 2.4–54.4 ± 9.1 pg/mg protein) (*p* < 0.001) (Figure 8a), IL-6 (72.4 ± 9.6–256.7 ± 9.3 pg/mg protein) (*p* < 0.001) (Figure 8b), MPO (12.6 ± 2.6–141.4 ± 12.8 U/mg of protein) (*p* < 0.001) (Figure 8d), and caspase-3 (9.9 ± 2–51.4 ± 2.6 ng/mg protein) (*p* < 0.001) (Figure 8e) were increased; however, the sciatic level of IL-10 (189.1 ± 14.6–46.6 ± 7.8 pg/mg protein) (*p* < 0.001) (Figure 8c) was decreased in the V groups on day 21.

The increases in TNF-α, IL-6, MPO, and caspase-3 levels and decrease in IL-10 levels in the V groups were inhibited by the LT100 mg/kg treatment (TNF-α: 54.4 ± 9.1–40.1 ± 3.9 pg/mg protein (−33%); IL-6: 256.7 ± 9.3–198.1 ± 12 pg/mg protein (−32%); IL-10: 46.6 ± 7.8–101.6 ± 10.8 pg/mg protein (39%); MPO: 141.4 ± 12.8–94.6 ± 11.6 U/mg protein (−36%); caspase-3: 51.4 ± 2.6–39.6 ± 3.5 ng/mg protein (−28%)) (V vs. V + T100, TNF-α: *p* < 0.001; IL-6: *p* < 0.001; IL-10: *p* < 0.001; MPO: *p* < 0.001; caspase-3: *p* < 0.001) and 300 mg/kg treatment (TNF-α: 54.4 ± 9.1–32.3 ± 4.2 pg/mg protein (−51%); IL-6: 256.7 ± 9.3–156.1 ± 12.7 pg/mg protein (−55%); IL-10: 46.6 ± 7.8–129.9 ± 14 pg/mg protein (58%); MPO: 141.4 ± 12.8–70.4 ± 10.5 U/mg protein (−55%); caspase-3: 51.4 ± 2.6–30.9 ± 3.5 ng/mg protein (−49%)) (V vs. V + T300, TNF-α: *p* < 0.001; IL-6: *p* < 0.001; IL-10: *p* < 0.001; MPO: *p* < 0.001; caspase-3: *p* < 0.001). These results indicate that the LT treatment inhibited VCR-induced increases in sciatic neuroinflammatory activity, as well as apoptosis markers.

## 4. Discussion

Based on this study, LT was found to protect against various VCR-induced pathologies, such as hyperalgesia, allodynia, oxidative damage, calcium dysregulation, neuroinflammation, and activation of the apoptotic pathway. This study was the first to demonstrate the potential of LT in mitigating VCR-induced pathophysiological dysfunction, possibly through antioxidative, calcium homeostasis, anti-inflammatory, and anti-apoptotic mechanisms, which could prove beneficial in treating chemotherapy-related peripheral neuropathy. Animal studies using the VCR-induced neuropathy model can be used to identify new treatments for chemotherapeutic-agent-associated neuropsychiatric disorders, as the behavioral alterations observed in rodents are similar to those seen in humans [6]. On day 7, VCR-induced neuropathy resulted in significant hyperalgesia, allodynia, and functional loss in animals, with the best nociceptive threshold being detected on day 14, which is consistent with earlier findings [5]. A-δ and C fiber neurons are believed to mediate the earliest nociceptive response to noxious mechanical stimuli [33,34].

Research indicated that VCR sensitizes dorsal horn nociceptive neurons, causing hyperalgesia and allodynia by inducing hyperresponsiveness in these neurons [5,6,35]. In vitro and in vivo studies also demonstrated that VCR damages Schwann cells and dorsal root ganglion (DRG) neurons by lowering laminin and neurite reflection, thereby limiting myelin formation. The release of inflammatory cytokines by damaged Schwann cells leads to neuropathic inflammation, which results in peripheral neuropathy, including hyperalgesia and allodynia, as well as nerve loss [36,37]. Following VCR administration, LT (at doses of 100 or 300 mg/kg/day, i.p.) was able to effectively reduce hyperalgesia and allodynia, while also increasing the MNCV and SNCV. These findings suggest that LT therapy may reduce the extent of neuropathy caused by VCR injections.

Our findings indicate that calcium accumulation within cells increased neuronal oxidative stress in VCR-induced neuropathy [35]. This buildup can activate secondary messengers, such as calpain and calmodulin, which alter neuronal excitability and lead to axonal degeneration by affecting the stability of axonal cytoskeleton proteins, resulting in hyperalgesia and allodynia [35,38]. Mitochondrial alterations may also contribute to VCR-induced neurotoxicity, calcium changes, oxidative stress changes, and dying back neuropathy [39]. LT’s calcium homeostasis action, which binds to glutamate receptors to prevent an excess calcium increase and maintain mitochondrial functions [40], likely contributes to ameliorating VCR-induced neuropathy. Nitrosative and oxidative reactions may also play a role in neuropathy development, as evidenced by significant increases in nitrite and MDA levels in nerve tissue following VCR administration, along with decreased levels of GSH, SOD, and CAT [41,42].

Following the LT therapy, the VCR-induced neuropathy was reduced, possibly due to its antioxidant properties, which restored animals’ nitrosative and oxidative stress indicators. MPO catalyzes the reaction between H_2_O_2_ and chloride ions, resulting in hypochlorite, which interacts with polyunsaturated fatty acids and induces lipid peroxidation [37]. Neutrophils also contribute to oxidative stress by producing reactive oxygen species that oxidize lipids, proteins, and nucleic acids in injured sciatic nerve tissue [43]. Inflammatory mediators, such as TNF-α, IL-1, and IL-6, were shown in clinical and experimental research to contribute to neuropathy progression and maintenance [44]. TNF-α, which is a crucial mediator of inflammatory responses, activates several other cytokines, and IL-1β and IL-6 are potent mediators of inflammatory processes [45,46]. Previous studies showed that the administration of TNF-α and IL-6 induces allodynia and hyperalgesia in rats [47]. Treatment with IL-10 or TNF-α, IL-1, and IL-6 receptor antagonists substantially mitigates allodynia and hyperalgesia in neuropathy animal models [48,49].

Following the VCR injection, the levels of TNF-α, IL-1, IL-6, and MPO were found to be significantly elevated in the nerve tissue of rats, while the IL-10 levels were lower, which is consistent with prior research [42,43]. This suggests that the inflammatory response in rats played a role in the formation of VCR-induced neuropathy. However, treatment with LT after the VCR administration led to a significant reduction in the TNF-α, IL-6, and MPO levels, and an increase in the IL-10 levels in rats. This indicates that LT had anti-inflammatory properties, which is consistent with previous research [11,12,14,50]. Therefore, the ameliorative effects of LT on VCR-induced neuropathy were likely due to its anti-inflammatory effect.

Excessive nitrosative and oxidative stress can activate the inflammatory response and release inflammatory mediators that initiate the apoptotic pathway, which was suggested as a key mechanism for neuronal cell death and a critical pathway involved in the progression of neuropathy development [3,4,5,6,7,8]. After a VCR injection, cells are damaged, oxidative/nitrosative stress is induced, inflammatory mediators are induced, and apoptosis is induced. This leads to extensive neuronal death in nerve tissue, which is believed to result in long-term behavioral impairments. Caspase-3 was found to be increased following the VCR administration, indicating that apoptotic cascades were initiated.

It has long been believed that neuropathy is associated with abnormal neuronal activity in the sciatic nerve [41]. LT’s anti-apoptotic effect on VCR-induced neuropathy can be attributed to its ability to reduce caspase-3 in the nerve tissue of rats treated with LT after a VCR injection [51]. In both animal and clinical studies, LT and cystine were effective in preventing oxaliplatin-induced peripheral neuropathy, mainly because they can promote the synthesis of GSH, which is a potential substance to prevent neuropathic pain [52,53]. LT also showed strong antioxidative and anti-inflammatory properties [12,54], suggesting that it may attenuate VCR-induced oxidative damage, neuroinflammation, and apoptosis. In addition, LT was found to ameliorate chronic constriction-injury-induced neuropathic pain [14]. In this study, it was discovered that LT reduced the VCR-increased levels of MDA, NO, MPO, total calcium, TNF-α, IL-1β, IL-6, and caspase-3 in the rat sciatic nerve while increasing VCR-reduced GSH, SOD, and CAT and IL-10 levels in the rat sciatic nerve. This suggests that the multiple functions of LT are related to several pathophysiological pathways. Based on the behavioral analyses conducted in this study, it is likely that these properties of LT contributed to its ability to ameliorate VCR-induced neuropathy.

## 5. Conclusions

**Figure 9:**
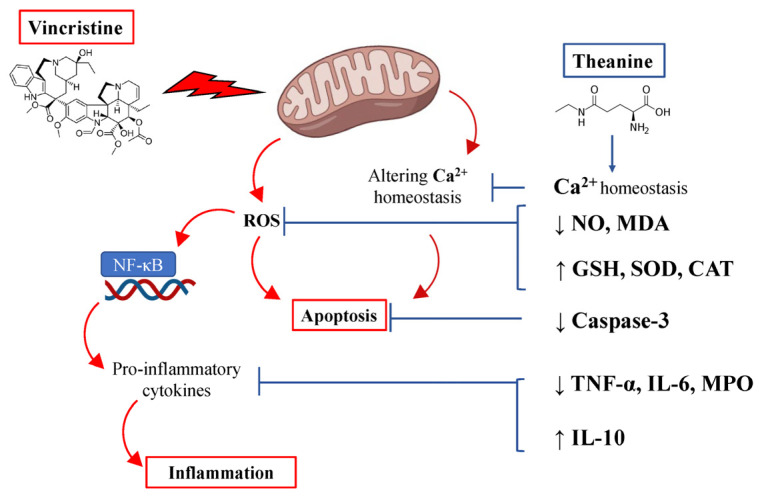
A schematic depiction of the protective potential of LT against VCR-induced neuropathy in rats. VCR increased the levels of MDA, NO, MPO, Ca^2+^, TNF-α, IL-1β, IL-6, and caspase-3, and reduced the GSH, SOD, CAT, and IL-10 levels in rat sciatic nerves. In this study, LT was found to significantly reduce VCR-induced oxidative stress, increase antioxidative strength, and reduce neuroinflammatory activity and apoptosis markers against VCR-induced neuropathy.

The results of this study validate chemotherapy-induced neuropathy pharmacologically in a relatively broad sense. Nevertheless, more research is needed to determine its exact mechanism of action and full potential as an analgesic. The role of LT in treating clinically relevant human neuropathy and its benefits should be investigated in the future. In this study, the protective effects of LT might be explained by its antioxidant, calcium homeostasis, anti-inflammatory, anti-apoptotic, and neuroprotective properties (Figure 9).

## Data Availability

The datasets used and/or analyzed in the current study are available from the corresponding author on reasonable request.
